# Single cell spatial profiling of FFPE splenic tissue from a humanized mouse model of HIV infection

**DOI:** 10.1186/s40364-024-00658-x

**Published:** 2024-10-08

**Authors:** Guoxin Wu, Samuel H. Keller, Luca Sardo, Brian Magliaro, Paul Zuck, Carl J. Balibar, Claire Williams, Liuliu Pan, Mark Gregory, Kathy Ton, Jill Maxwell, Carol Cheney, Tom Rush, Bonnie J. Howell

**Affiliations:** 1 MRL, Merck & Co., Inc, Rahway, NJ USA; 2 NanoString Technologies, a Bruker Company, Seattle, WA USA

**Keywords:** FFPE, CosMx SMI, HIV, Single cell, Transcriptomic profiling, SYK, qPCR, Biomarker

## Abstract

**Background:**

Latency remains a major obstacle to finding a cure for HIV despite the availability of antiretroviral therapy. Due to virus dormancy, limited biomarkers are available to identify latent HIV-infected cells. Profiling of individual HIV-infected cells is needed to explore potential latency biomarkers and to study the mechanisms of persistence that maintain the HIV reservoir.

**Methods:**

Single cell spatial transcriptomic characterization using the CosMx Spatial Molecular Imager platform was conducted to analyze HIV-infected cells in formalin-fixed paraffin-embedded sections of splenic tissue surgically obtained from an HIV-infected humanized mouse model. Regulation of over a thousand human genes was quantified in both viremic and aviremic specimens. In addition, in situ hybridization and immunohistochemistry were performed in parallel to identify HIV viral RNA- and p24-containing cells, respectively. Finally, initial findings from CosMx gene profiling were confirmed by isolating RNA from CD4 + T cells obtained from a person living with HIV on antiretroviral therapy following either PMA/Ionomycin or DMSO treatment. RNA was quantified using qPCR for a panel of targeted human host genes.

**Results:**

Supervised cell typing revealed that most of the HIV-infected cells in the mouse spleen sections were differentiated CD4 + T cells. A significantly higher number of infected cells, 2781 (1.61%) in comparison to 112 (0.06%), and total HIV transcripts per infected cell were observed in viremic samples compared to aviremic samples, respectively, which was consistent with the data obtained from ISH and IHC. Notably, the expression of 55 genes was different in infected cells within tissue from aviremic animals compared to viremic. In particular, both spleen tyrosine kinase (*SYK*) and *CXCL17*, were expressed approximately 100-fold higher. This data was further evaluated against bulk RNA isolated from HIV-infected human primary CD4 + T cells. A nearly 6-fold higher expression of *SYK* mRNA was observed in DMSO-treated CD4 + T cells compared to those stimulated with PMA/Ionomycin.

**Conclusion:**

This study found that the CosMx SMI platform is valuable for assessing HIV infection and providing insights into host biomarkers associated with HIV reservoirs. Higher relative expression of the *SYK* gene in aviremic-infected cells from the humanized mouse HIV model was consistent with levels found in CD4 + T cells of aviremic donors.

## Introduction

Despite the advancements of antiretroviral therapies (ART), HIV remains incurable and persists in tissue reservoirs [1–3]. Some of these reservoirs remain partially active even during ART, leading to the transcription of viral RNA and production of viral proteins. These transcriptionally and translationally competent cells are believed to contribute to viral rebound when ART is discontinued [4, 5]. Currently, our understanding of the tissue microenvironments that enable the establishment and maintenance of HIV latency in infected cells remains limited [6–8]. Additionally, difficulty in detecting viral RNA and protein expression from integrated provirus makes it challenging to identify biomarkers of HIV persistence and fully understand virus host interactions.

To expand our knowledge of the mechanisms underlying HIV latency, it is crucial to understand the spatial distribution of RNA and protein profiles among infected cells, the communication between infected and uninfected cells, and the influence of the microenvironment upon infected tissues. The development of technologies such as single-cell RNA sequencing (scRNA-seq: 10x Genomics) has provided an opportunity to profile RNA at a high-plex level with single-cell resolution [9, 10]. However, this technology requires tissue dissociation and single-cell isolation which disrupts the preservation of spatially distributed information throughout infected tissues. Other techniques, such as in situ hybridization (ISH) and immunohistochemistry (IHC), can evaluate whole fixed tissue samples and are commonly used to assess the spatial distribution of RNA and proteins, respectively [11, 12]. Although specific, these methods have limitations in detecting subcellular expression and the number of targets they can identify, making them unsuitable for high-plex multiomic analyses.

Recent advancements in single-cell spatial transcriptomics (SCST) technology offer advantages for studying the subcellular spatial distribution of RNA and protein in tissues [13, 14]. Spatial molecular imaging (SMI) allows the measurement, through probe counts, of RNAs and proteins at subcellular resolution in intact tissue sections using multiple cycles of nucleic acid hybridization with fluorescent molecular barcodes. Two main platforms, CosMx™ SMI by NanoString Technologies and Xenium by 10x Genomics, enable multiomic assessment by means of multiplex staining at single-cell and subcellular resolution on formalin-fixed paraffin-embedded (FFPE) sections. [15–18]. These SMI technologies are automated on integrated platforms that utilize different staining chemistries. Although promising, they are still in the early stages of validation.

In this study, we utilized a novel high-plex RNA CosMx SMI to characterize FFPE mouse spleen sections containing HIV-infected human cells from a humanized mouse model. Our goal was to determine whether CosMx SMI could detect HIV RNA and to validate findings with ISH. Additionally, we aimed to investigate potential changes in RNA profiling among infected human cells by using a targeted 1000-plex human RNA probe panel. We found that CosMx SMI successfully detected differences in both HIV-infected cells and HIV transcripts per infected cell between tissue samples obtained from viremic and aviremic animals. Notably, we identified significantly higher expression levels of specific genes, particularly the *SYK* gene, in HIV-infected cells from aviremic compared to viremic. These findings were further confirmed in vitro using aviremic human HIV-infected CD4 + T cells following stimulation with PMA/Ionomycin. In addition, the CosMx SMI enables differential expression and characterization of tissue surrounding infected cells which were not utilized due to the chimeric nature of the humanized mouse model. Thus, the CosMx SMI technology holds the potential to explore novel biomarkers from HIV-infected host cells and aid in better understanding the mechanisms underlying HIV latency.

## Materials and methods

### Mouse HIV model and tissue sections preparation

Fresh human peripheral blood mononuclear cells (PBMCs) were obtained from Biological Specialty Company (Colmar, PA). PBMCs were then activated with Phytohemagglutinin (PHA) at a concentration of 5 µg/ml in complete RPMI-1640 (cRPMI) medium with 10% fetal bovine serum (FBS) and 20 U/ml Interleukin-2 (IL-2) for 3 days. After activation, the PBMCs were infected with R8 wild-type replication-competent HIV virus. The multiplicity of infection (MOI) used was 0.1, and the infection process lasted for 4 h. Following infection, the tubes were centrifuged at 800 x g for 5 min, and the cells were washed once with RPMI-1640 medium. The infected PBMCs were then cultured in T175 flasks with fresh cRPMI. One day post-infection, the cultured PBMCs were collected and resuspended at a concentration of 150 × 10^6^ PBMCs/ml in phosphate-buffered saline (PBS). Immunodeficient NOD-SCID-IL-2ry-/- mice were injected intraperitoneally (IP) with 0.2 ml infected PBMCs (30 × 10^6^ cells/mouse). The plasma viral load in mice was measured weekly using quantitative PCR (qPCR) after retro-orbital bleeding. Starting from week 5, the antiviral integrase inhibitor raltegravir (RAL) [19] was administered to the mice. The loading dose of RAL was 50 mpk orally on Day 0, followed by a maintenance dose of 100 mpk in chow food. A control group of mice received the same chow without RAL. Mouse blood was collected on day 1, 3, and 7 post-dosing to monitor the effects of RAL administration. In addition, spleens were collected at the end of week 4 post-ART from both viremic mice with vehicle dosing and aviremic mice with undetectable plasma viral load. Tissue was fixed with 4% paraformaldehyde (PFA) at room temperature (RT) for 48 h. The fixed spleens were then embedded in paraffin wax, and 5 μm thick FFPE sections were cut using a microtome. Tissue sections were mounted onto positively charged glass slides and stored at -20 °C for subsequent staining and analysis in ISH, IHC, and CosMx SMI within 1 month after sectioning.

### Tissue immunohistochemistry

Mouse spleen tissue FFPE sections were first stained with IHC on a Leica Bond RX autostainer (Leica Biosystems) [11] with an antibody specific to HIV p24 protein to locate HIV-infected human cells within the tissue section. BOND Polymer Refine Red Detection kit (Cat# DS9390) was obtained from Leica Biosystems. Heat- induced epitope retrieval was performed in buffer ER1 for 20 min. To increase assay sensitivity, two anti-p24 antibodies were obtained from Capricorn (Cat# HIV-018-48304) and ZeptoMetrix (Cat# 801136) and combined during antibody staining. Each antibody was prepared with Da Vinci Green Diluent buffer (Cat# PD900M, BioCARE Medical) at a final concentration of 3 µg/ml. Alkaline phosphatase (AP)-labeled anti-mouse IgG detection antibody and AP red substrate were included in the kit. All staining procedures were performed according to manufacturer’s instructions with an automated IHC protocol. After staining, slides were washed once with ddH_2_O for 1 min, then dehydrated with HistoPrep 100% denatured ethyl alcohol (Fisherbrand, HC8001GAL) with two soaks (5 min each) followed by two soaks with HistoPrep xylene (Fisherbrand, HC7001Gal) (5 min each). Slides were then mounted with one drop of EcoMount (BioCARE Medical, EM897L), coverslip added and dried at RT overnight. Slide images were acquired using an Aperio scanner (Leica Biosystems) and analyzed with digital pathology imaging software (HALO, Indica Labs).

### Tissue section ISH

ACD RNAscope™ 2.5 LS Reagent Kit – RED [11, 12] was used to stain for HIV viral RNA in FFPE mouse spleen sections. The FFPE mouse spleen sections were first dewaxed and enzymatically digested using an automated Leica Bond RX system. RNA probe staining was performed according to the manufacturer’s instructions on the Leica Bond RX system. For HIV detection, a specific RNA probe targeting the complete sequence of the HIV-1 virus was used (RNAscope™ 2.5 LS Probe-V-HIV1-CladeB: ACDBio Cat#416118). After in situ hybridization of the probe, AP substrate developer from the above red detection kit was applied to visualize the location of HIV RNA in the tissue. Subsequently, the slides were washed with ddH_2_O, sequentially dehydrated with 100% ethanol and xylene, and finally mounted with EcoMount.

### Tissue CosMx spatial molecular imaging (SMI)

Once the presence of viral markers and quality of the tissue sections were confirmed with ISH and IHC, adjacent mouse spleen sections were used for NanoString CosMx SMI staining. The RNA target readout on the CosMx SMI instrument was performed according to the method previously described [13, 14]. The slides from both viremic and aviremic mouse spleen FFPE sections were loaded onto the NanoString CosMx SMI instrument. Slides were stained with specific antibodies (anti-CD3, CD45, PanCK, B2M/CD298) and DAPI to visualize the cell morphology and select the field of view (FOV). FOV selection was guided by tissue cell morphology markers to ensure that similar tissue structures were captured in both viremic and aviremic sections. A total of 40 FOVs were placed on the tissue to match the regions of interest identified by ISH from an adjacent section. After FOV selection, slides were pre-treated with proteinase K and then hybridized with ∼ 1000-plex human mRNA probes and custom-designed probes specific to HIV *gag*, *pol*, *env*, *nef* and *vif* sequences. The mRNA probes allowed for the detection of specific mRNA targets in tissue sections. A probe targeting the human heterogeneous nuclear ribonucleoprotein K (HNRNPK) was used as a control to identify human cells in the mouse tissue section. Following probe hybridization, unbound probes were washed away, and imaging buffer was added to the slides prior to imaging. The photocleavable linkers on the fluorophores of the reporter probes were then released by UV illumination, followed by washing with strip wash buffer. The fluorescent barcodes were read subsequently by CosMx imager and decoded by probe identity to generate in-situ CosMx probe detection data [13–15]. The four immunofluorescent antibodies and DAPI were used to segment tissues into cells using a machine learning algorithm [20, 21]. Target transcripts were assigned to cells and subcellular compartments based on segmentation boundaries.

Target counts were then normalized by taking total number of counts of a gene within a cell and dividing by total number of counts across all genes within that same cell and multiplying by the average counts per cell across the entire dataset. UMAP dimensionality reduction used normalized, square root transformed expression values for all genes. Semi-supervised cell typing utilized the InSituType package [22], comparing cell profiles to the default package-contained immune profiles with a negative binomial model and allowing for the identification of up to six novel clusters. Manual verification of cell type annotations examined known markers of gene expression, protein expression, and location in the tissue. A negative-binomial model was used to assess whether each gene was differentially expressed between groups, regressing raw counts for each target against group using the R package “Nebula” [13]. The model included an offset term for each cell’s total counts and the total expression of the target gene in neighboring cells of other types within 0.05 mm as a fixed effect in the regression model. The goal of this fixed effect is to reduce the impact of potential minor cell segmentation errors which naturally arise in image-based spatial transcriptomic technologies.

### Quantitative reverse transcription polymerase chain reaction (qPCR)

PBMCs were isolated from HIV-infected ART-suppressed subjects using either leukapheresis or whole blood via Ficoll-gradient centrifugation, obtained from Thomas Jefferson University (TJU). CD4 + T cells were then isolated from PBMCs using negative selection with the EasySep Kit (STEMCELL Technologies, Cat# 17952). The CD4 + T cells were treated with either 0.1% DMSO or a combination of 100 ng/ml final concentration phorbol 12-myristate 13-acetate (PMA) and 1 µg/ml ionomycin in culture medium for a duration of 48 h. The cells and culture medium were collected by centrifugation, and RNA was isolated from the cell pellet using the RNeasy kit (Qiagen, Cat# 74104). Quantitative PCR (qPCR) was performed using the TaqMan Fast Virus 1-Step Master Mix from Thermo-Fisher (Cat#4444434). A total of 2 µl of purified RNA was used as the template for qPCR [23]. Gene expression assays for the primers/probe specific to human *CXCL17* (Hs01650998_m1), SYK (Hs00895377_m1), CD4 (Hs01058407_m1), S100A4 (Hs00243202_m1), and HIV integrase [24] were obtained from Thermo-Fisher. The qPCR was conducted using the QuantStudio 12 K Flex system. Ct values, which indicate the cycle threshold, were obtained after reverse transcription and 40 cycles of PCR [23]. The fold-change for mRNA expression of HIV integrase, *CXCL17*, *SYK*, *CD4*, and *S100A4* following PMA/ionomycin stimulation was calculated by using either human *CD4* or *S100A4* mRNA Ct value as a reference for adjustment.

### Plasma viral load measurement

Blood samples were collected into EDTA Minicollect tubes from mice through retro-orbital bleed following the injection of HIV-infected human PBMCs and administration of the anti-viral compound raltegravir (RAL). Plasma was obtained by centrifuging the blood at 4000 x g for 10 min at 4^o^C. RNA was isolated using MagMax-96 Viral RNA Isolation Kit (ThermoFisher, Cat #AM1836). Quantitative PCR (qPCR) was performed using HIV integrase and *gag* Taqman primers/probe, similar to the aforementioned procedure. To determine the copy number, HIV standard RNA was extracted from the MoltIIIb cell line [25] and quantitated using QuantStudio 3D digital real-time PCR system. Based on the standard curve, the plasma HIV viral load (copies/ml) was calculated.

### HIV p24 Simoa

Supernatant obtained from the cell culture was centrifuged at 10,000 x g for 5 min at RT to remove insoluble material before measuring p24 levels on the Quanterix analyzer. The assay reagents and reaction conditions for the p24 measurement followed the protocol provided by the Quanterix manufacturer’s p24 kit, as previously described [26–28]. The concentration of p24 was determined using a calibration curve fitted with a 4-parameter curve fitting method.

### Statistical analysis

Quantitative data analysis was performed using GraphPad Prism 10 (GraphPad Software, Inc., California). The data were presented as means ± SEM. A significance level of *p < 0.05, **p < 0.01, ***p < 0.001 and ****p < 0.0001 was used in this study. Statistical significance was determined using either Tukey-Kramer ANOVA or Student’s t-tests.

## Results

### Characterization of mouse spleen FFPE tissue section in ISH and IHC

**Fig. 1:**
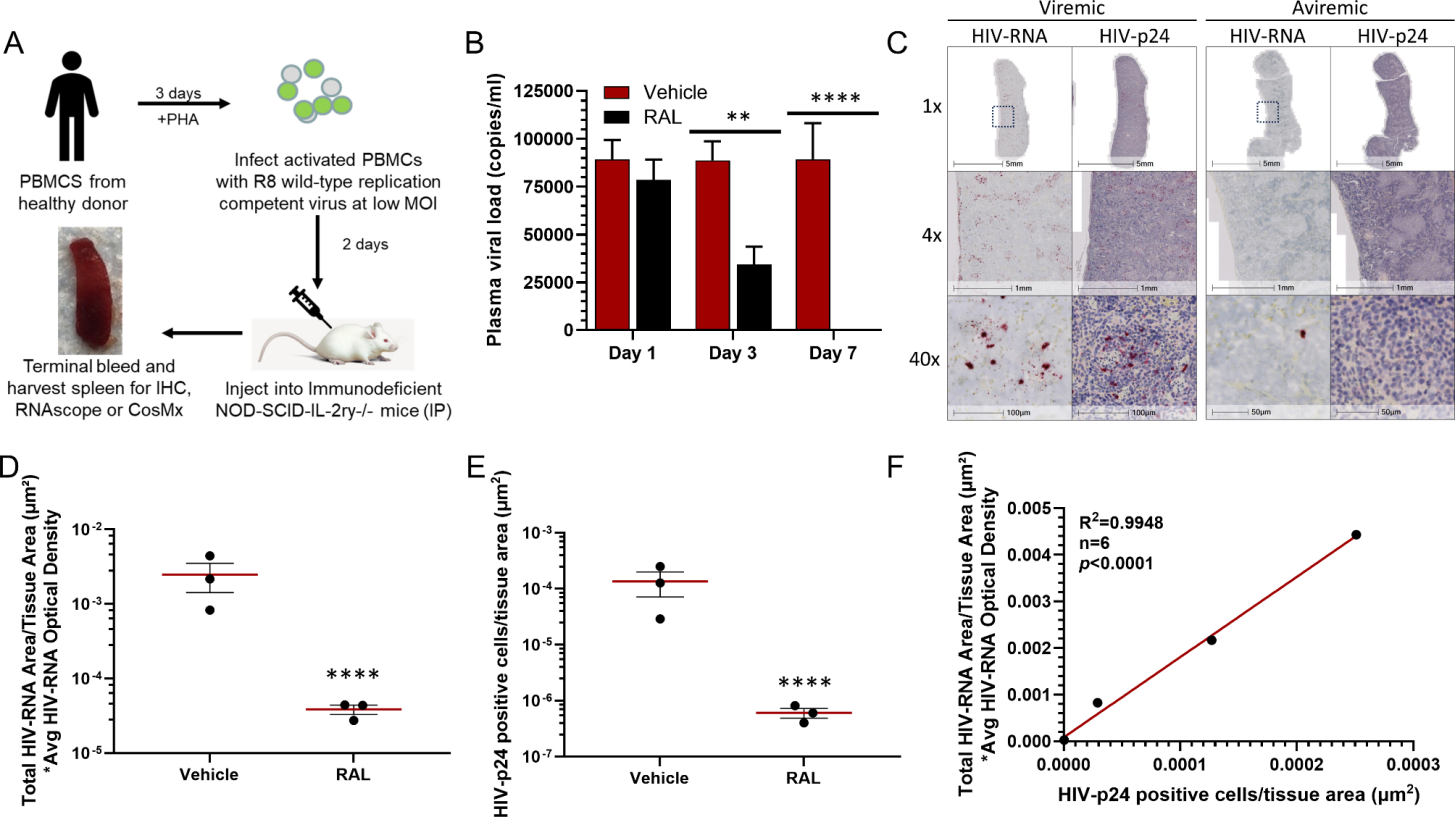
Characterization of humanized mouse HIV model: **(A)** Generation of HIV model in immunodeficient NOD-SCID-IL-2ry-/- mice with IP injection of HIV- infected human PBMC. **(B)** Plasma viral load (copies/ml) in vehicle and anti-viral integrase RAL-treated mice. **(C)** Imaging of viral RNA (RNAscope) and HIV p24 protein (IHC in FFPE spleen section) from HIV-infected vehicle treated (viremic) and anti-viral treated (aviremic) mice. **(D)** Quantitation of HIV viral RNA-containing cells in images from vehicle and RAL-treated mice. **(E)** Quantitation of HIV p24 protein-containing cells in tissues from vehicle and RAL-treated mice. **(F)** Correlation between tissue HIV viral RNA- and p24-containing cells in vehicle and anti-viral RAL-treated mice (*n* = 6)

To evaluate the applicability of the CosMx single-cell SMI tool for subcellular mRNA and protein profiling of HIV + tissue samples, a humanized mouse HIV model was developed as a proof of concept in this study (Fig. 1). The schematic procedure for generating the humanized mouse HIV model is shown in Fig. 1A, where replication-competent HIV virus was used to infect PHA-activated human PBMCs in vitro, and the infected cells were then injected intraperitoneally into immunodeficient mice. Weekly monitoring of the viral load in plasma revealed that viremia reached a plateau at 3 weeks post-injection, with a plasma viral load of approximately 1 × 10^5^ copies/ml in all mice (*n* = 6). The viral load remained stable at week 5. To assess the effect of anti-viral treatment, 5 weeks post-injection, three of the six mice were administered RAL daily through chow food. The results, shown in Fig. 1B, demonstrated a decrease in viral load at day 3 post-dosing, and no detectable viral load in plasma at day 7, mimicking a condition of aviremia. In contrast, there was minimal change in plasma viral load observed in the three vehicle-treated mice that were then used in this model to simulate a viremic condition. For simplicity, the animals and relative biological specimens (i.e. blood, tissue) are referred to as viremic or aviremic, respectively. At the end of week 4 post-dosing, the mouse spleens were surgically isolated. The spleens were then fixed with PFA and paraffin-embedded. FFPE sections were obtained from both viremic and aviremic mice. Blood samples from viremic mice displayed plasma viral loads of 89,333 ± 18,877 copies/mL, while specimens from aviremic animals had plasma viral loads of less than 9 ± 10 copies/mL.

**Fig. 2:**
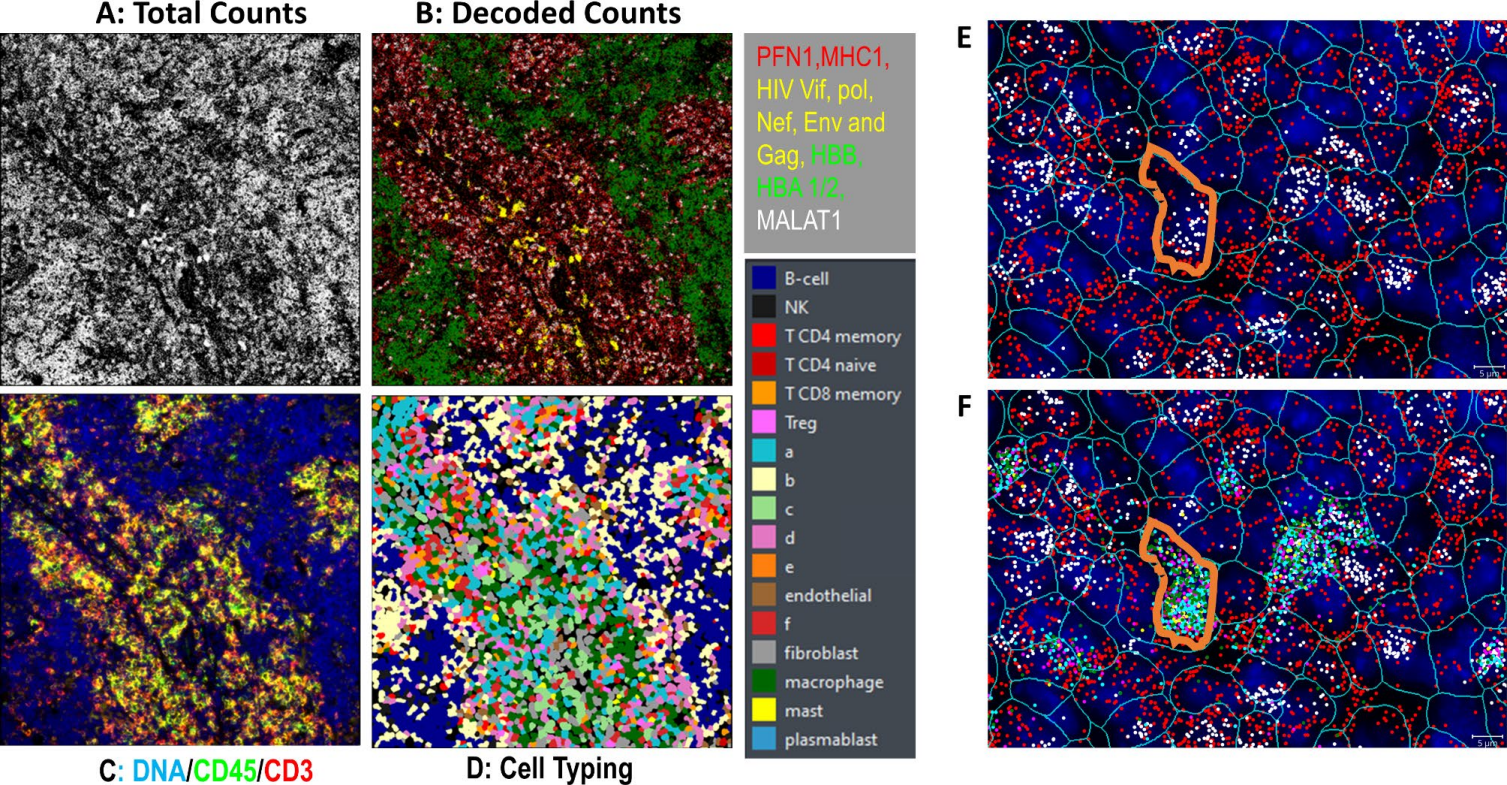
CosMx SMI technology: (**A**) CosMx SMI raw data image representing total counts collected from 10 probes, (**B**) CosMx SMI raw data image representing decoded counts collected from 10 probes, (**C**) Image of CosMx morphology marker staining using anti-CD3 and anti-CD45 antibodies and DAPI, (**D**) CosMx cell typing result, (**E**) Individual cell cytoplasm and nucleus localization, and (**F**) HIV RNA located within an individual infected cell’s cytoplasm. Cell border is highlighted in orange

The spleens were then cut into 5 micrometer thick slices and stained to assess the distribution of viral markers throughout the tissue. ISH with a viral RNA-specific probe demonstrated the presence of HIV- positive cells randomly distributed throughout the white pulp regions within the spleen from sagittal sections (1x magnification) (Fig. 1C). Additionally, HIV p24 protein was stained using anti-p24 antibodies, and displayed a distribution pattern similar to that of the viral RNA (Figs. 1C and 2x magnification). In contrast, only a few positive cells in both ISH and IHC stains were detected in the aviremic mice spleens with a random distribution in the white pulp (40x magnification) (Fig. 1C). Compared to the vehicle-treated spleen sections, both viral RNA (Fig. 1D) and p24 protein (Fig. 1E) levels were significantly decreased in the RAL-treated group. The patterns of viral RNA and p24 protein detection following RAL treatment were similar and displayed a correlation with an R^2^ value of 0.995 (*p* < 0.0001) (Fig. 1F). These findings suggest that the antiretroviral treatment effectively decreased both plasma viral loads and viral RNA and p24 detection in splenic tissue in this humanized mouse HIV model. The viral RNA and protein within the tissue FFPE sections remained intact, making these sections suitable for CosMx transcriptomic profiling.

### CosMx SMI overview

The application of CosMx SMI for subcellular mRNA profiling was evaluated using a 1000-plex human RNA panel and then applied to samples obtained from both viremic and aviremic mice. The composition of the 1000-probe panel included 243 genes for cell typing, 269 genes for cell function, 435 genes for cell-cell interaction, and 46 genes for hormone activity. Additionally, 5 custom-designed HIV probes targeting HIV *gag*, *pol*, *env*, *nef* and *vif* regions were added to the panel, as well as one human-specific probe targeting HNRNPK to help distinguish human from mouse cells. First, the quality of the CosMx SMI staining for both viremic and aviremic tissue sections was determined (Table 1). Both tissue sections had low background levels, as characterized by low rates of nonspecific binding, negative probes, and false codes. As shown in Table 1, the mean number of transcripts per cell was 294 for aviremic slides and 268 for viremic slides, with an average of over 100 transcripts per cell (as a threshold criteria) among 40 FOV. The probe detection sensitivity of the CosMx SMI was determined to be 1 to 2 copies per cell.

**Table 1:** CosMx run overview for both viremic and aviremic tissue samples

	Aviremic mouse spleen	Viremic mouse spleen	
Number of FOVs	40	41	
Total tissue area (mm^2^)	9.64	9.86	
Mean single cell size (µm^2^)	55	57.2	
Number of cells in final analysis	175,219	172,242	
Total transcripts assigned to cells	51,507,981	46,166,341	
Mean transcripts per cell	294	268	
Maximum transcripts per cell	2561	2456	
Mean unique genes per cell	101	99	
Mean transcripts per µm^2^	5.3	4.7	
Mean negative per plex per cell	0.017	0.017	
Mean false codes per plex per cell	0.013	0.012	

The readout process of the CosMx SMI technology is shown in Fig. 2. As depicted in Fig. 2A, tissue imaging was not informative based only on the total counts from SMI. However, after decoding the bound probe barcodes, morphological, spatial, and cell-type specific information became evident. As in Fig. 2B, mRNA in specific areas can be highlighted, indicating spatial features within the tissue section. Among the colors represented, red indicates eukaryotic mRNA probes Profilin 1 (*PFN1*) and *MHC1*, yellow indicates 5 different HIV probes, green indicates alpha-hemoglobulin (*HBA1* and *HBA2*) and beta-hemoglobulin (*HBB*) mRNA and white indicates nucleus-localized *MALAT1* mRNA. Figure 2C displays tissue distribution of immune cells stained with anti-CD3 and anti-CD45 antibodies and includes DAPI for nuclear DNA staining. By combining mRNA distribution and composition among nucleated cells, cell typing was predicted (Fig. 2D). Individual colors were used (refer to Fig. 2D legend) to identify discrete cell types, with letter designations assigned to cell types that did not match the reference profile of cell type expression patterns used. Figure 2E and F show both the localization of host genes and HIV RNA in viremic spleen cells. Cell borders within the tissue context were outlined based on antibody staining of cell membranes; the outline of one cell is highlighted as an example (Fig. 2E). The locations of the cell’s nucleus and cytoplasm were identifiable based on the position of the cytoplasmic probes *PFN1* and *PPIA* and nuclear probes RNA *NEAT1* and *MALAT1* (Fig. 2E). Figure 2F combines the staining of HIV *gag*, *pol*, *env*, *nef* and *vif* with the genes seen in Fig. 2E and shows, depicted by the cell outlined in orange, that all HIV probes are elevated within this cell. These results exemplified the ability of CosMx SMI to detect cellular and sub-cellular multi-plex mRNA profiling by in situ hybridization and probe decoding to facilitate the identification of distinct cell types.

**Fig. 3:**
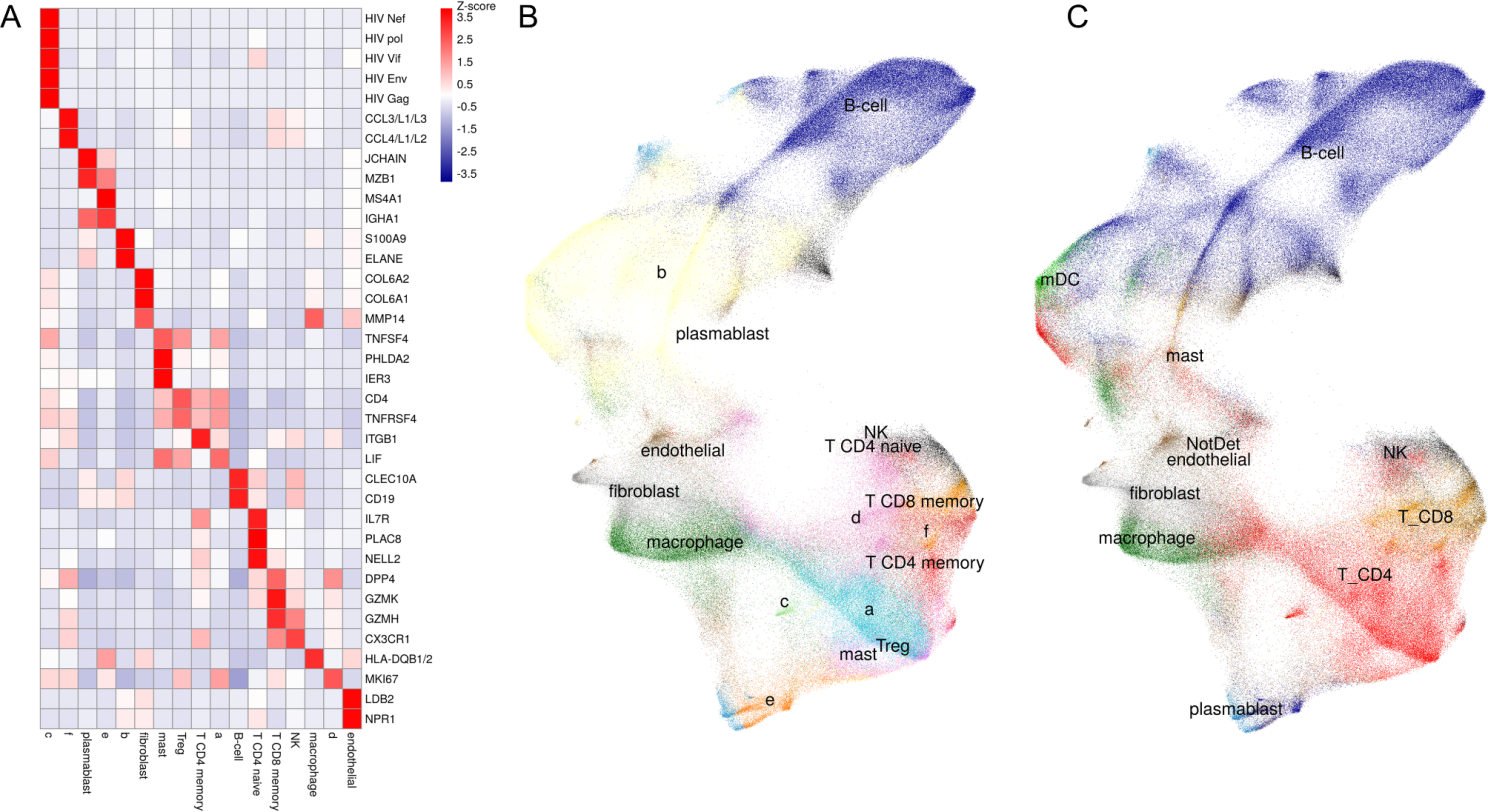
Gene Heatmap and UMAP cell clustering: (**A**) Genetic marker heatmap based on cell typing, (**B**) Semi-supervised cell clustering in UMAP, (**C**) Supervised UMAP cell clustering

To identify which PBMC subset was infected with HIV in the mouse spleen sections, semi-supervised clustering was applied to annotate all cells with either a known cell type or a novel cell type. With the goal of identifying as many of the human PBMC types as possible, an immune cell type reference profile was used. Cells grouped into novel cell types had consistent expression profiles within one cell type but did not match the expression profile of any known cell type in the initial reference profile. A gene heatmap was generated that showed the highest-scoring marker genes for each cell type and included all five HIV-specific probes that were predominantly located in type c cells and appeared at lower levels in CD4 + naïve T cells (Fig. 3A). Figure 3B and C illustrate cell clustering according to Uniform Manifold Approximation and Projection (UMAP). Semi-supervised cell clustering places the small group of type c cells close to the CD4 + memory T cell population (Fig. 3B). Supervised cell clustering, which forces all cells to be annotated as the closest cell type within the initial reference profile, confirms that type c is in fact a subset closely resembling CD4 + T cells (Fig. 3C), but slightly different from native CD4 + T cells. Based on this data, it can be predicted that the majority of HIV-infected cells in human PBMC are CD4 + T cells while other subsets of infected cells are less well- represented in the spleen section from a viremic animal. This qualitative data indicated that CosMx SMI technology can be applied to identify HIV-infected cell types based on mRNA profiling and semi-supervised cell typing.

### Measurement of HIV genes in FFPE sections in CosMx SMI

**Fig. 4:**
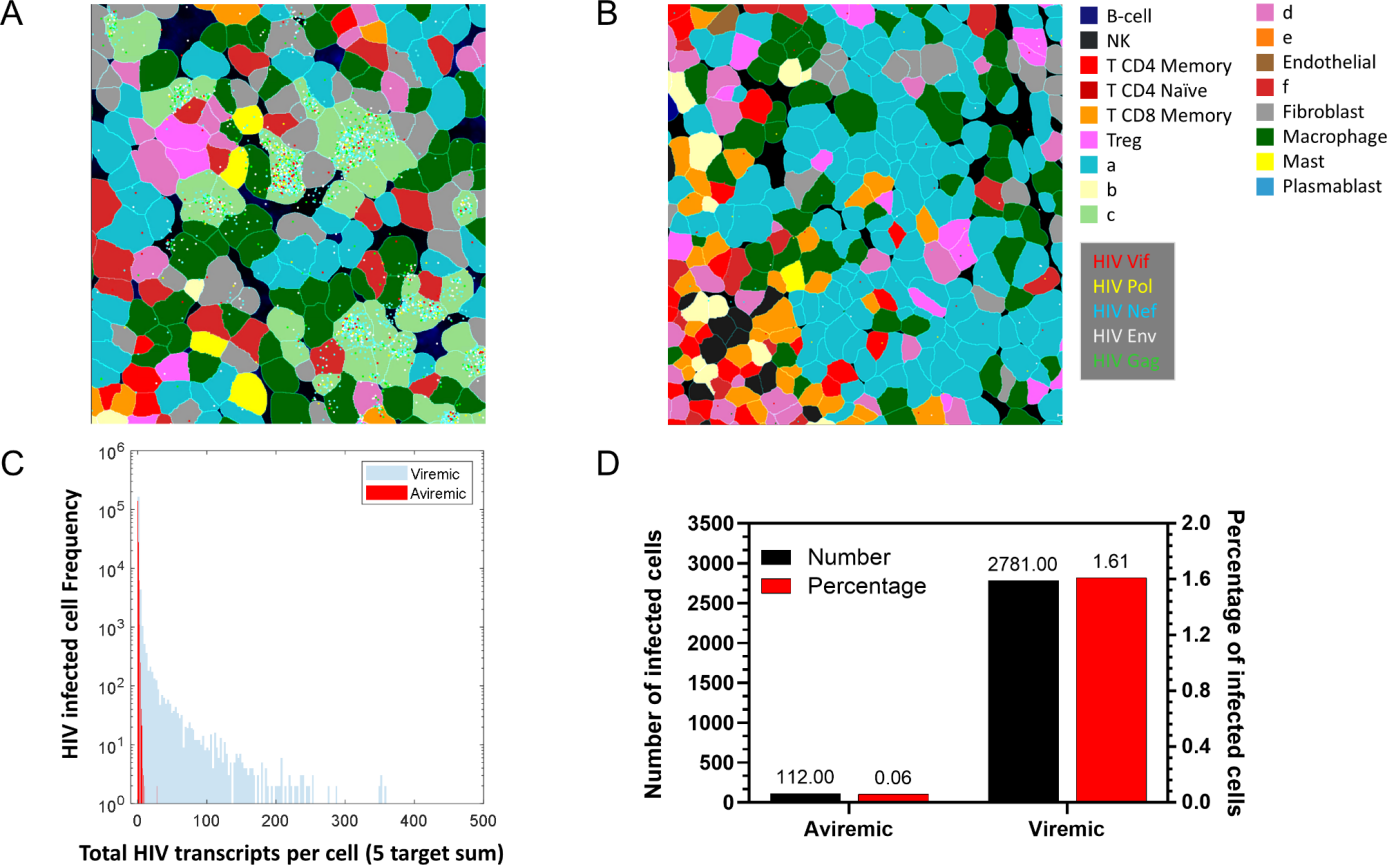
Characterization of HIV-infected cells in viremic and aviremic conditions: (**A**) CosMx cell typing image of HIV-infected cells in viremic section, (**B**) CosMx cell typing image of HIV-infected cells in aviremic section, (**C**) Correlation between number of total infected cells and HIV transcripts per infected cell in viremic vs. aviremic sections, (**D**) Comparison of HIV-infected cell number based on the criteria of 10 or more normalized copies per cell and percentage of infected cells based on the total analyzed cells between viremic and aviremic sections

Next, we sought to compare tissue sections obtained from aviremic and viremic mice to determine the differences in HIV-infected cell number and/or total HIV transcripts per infected cell (Fig. 4). As shown in Fig. 4A, HIV-infected cells were randomly distributed in viremic spleen tissue based on HIV probe location. All five probes targeting different HIV RNA regions hybridized well to the infected cells, suggesting that viral RNA was intact in the FFPE tissue section. As expected, the aviremic condition shown in Fig. 4B exhibited fewer HIV-infected cells when compared with that of Fig. 4A viremic section. Figure 4C shows the relationship between HIV-infected cell number compared to the total HIV transcripts per infected cell in both viremic and aviremic samples, grouping all five HIV targets together. Figure 4D compares the calculated total number and percentage of infected cells from 40 FOV between viremic and aviremic sections, based on total HIV transcripts per infected cell, using a threshold of 10 normalized HIV transcripts to define an HIV positive cell. There were only 112 HIV-infected cells (0.06% based on total number of cells analyzed) in the aviremic section to 2781 HIV-infected cells (1.61% based on total number of cells analyzed) in the viremic section. The data generated with CosMx SMI is consistent with ISH, showing a significant difference in the number of infected cells between viremic and aviremic sections. Notably, CosMx SMI detected the difference in total HIV transcripts per infected cell whereas ISH did not due to the limited subcellular resolution of the assay. Taken together, these data indicated that CosMx SMI staining of the humanized mouse HIV model worked well and can provide quantitative insights into the transcriptomic profile of an entire tissue section.

### Measurement of human genes in FFPE sections in CosMx SMI

**Fig. 5:**
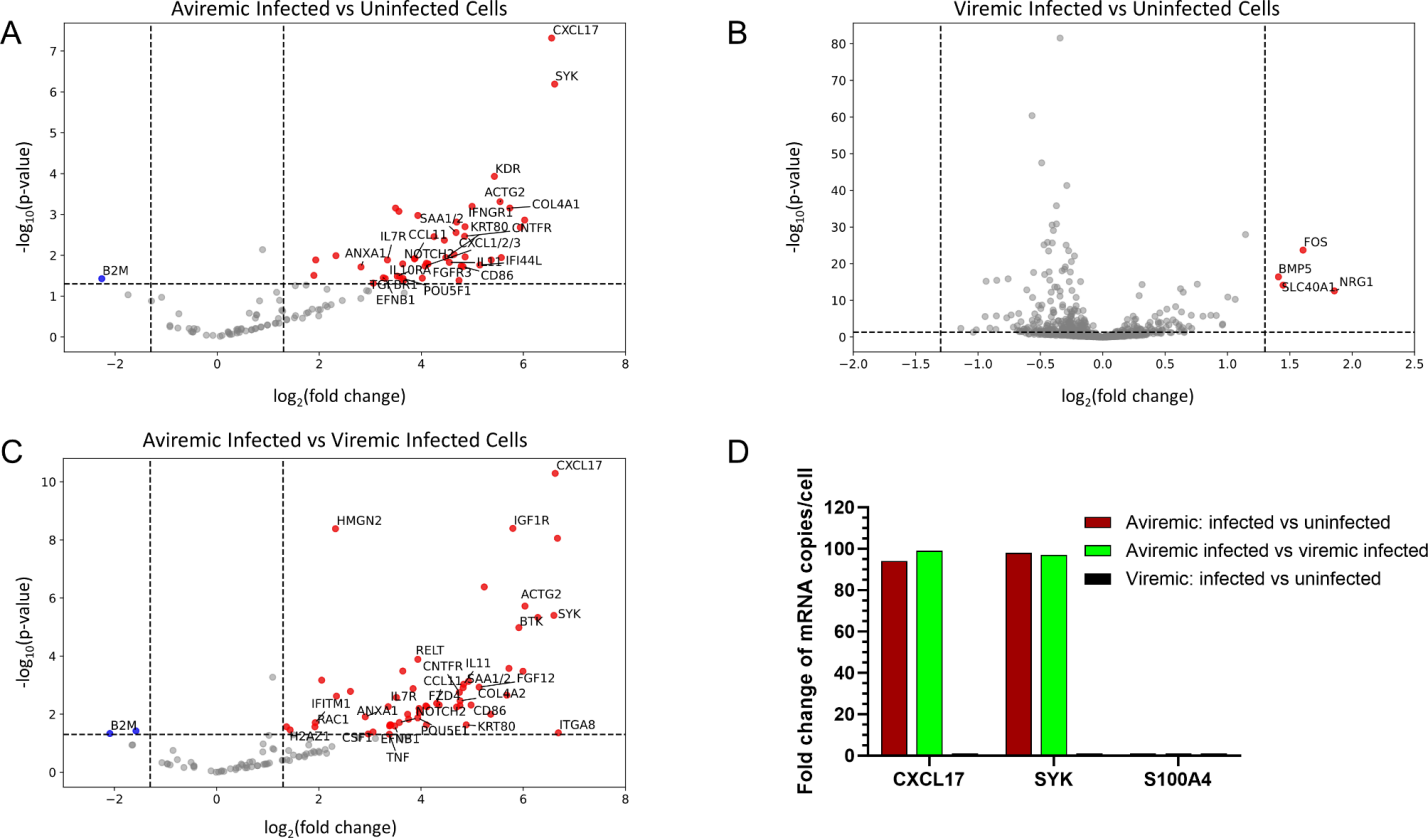
Comparison of 1000-panel human gene profiling between viremic and aviremic sections: **(A)** Gene expression of *SYK* and *CXCL-17* were upregulated significantly (*p* < 0.0001) in infected vs. uninfected cells under aviremic conditions, **(B)** Upregulation of genes in infected vs. HIV-uninfected CD4 + T cells under viremic conditions (*p* < 0.05), **(C)** Upregulation of genes in HIV-infected CD4 + T cells in aviremic section as compared with that of viremic section (*p* < 0.05). Several genes including *SYK* and *CXCL17* with *p* value < 0.0001, **(D)** Fold- change of *CXCL17*, *SYK* and *S100A4* genes among infected vs. uninfected cells in aviremic vs. viremic conditions

After obtaining proof of concept data from the CosMx SMI staining, we sought to evaluate whether any differences could be detected in the expression of 1000-plex human genes in the viremic and aviremic conditions. Comparing gene expression between HIV-infected and uninfected CD4 + T cells in the aviremic tissue section demonstrated that many human host genes were significantly upregulated in infected cells (Fig. 5A) (*p* < 0.05). The gene expression levels of *CXCL17* and *SYK* were particularly increased in infected cells compared to uninfected cells in the aviremic section. The ratio of gene expression in infected cells compared to that of uninfected cells was 97 and 98 for *CXCL17* and *SYK*, respectively, with *p* value lower than 10^− 4^ (*p* < 0.0001) (Fig. 5A). In the viremic section, multiple genes were also upregulated significantly (*p* < 0.05) in infected cells as compared with that of uninfected cells (Fig. 5B). However, the increased ratio in gene expression was less than 4- fold. Markedly, no significant difference in gene expression was observed for *CXCL17* and *SYK* between infected and uninfected cells within viremic sections (Fig. 5B). In the comparison of infected cells between aviremic and viremic sections, 55 genes were also upregulated significantly (*p* < 0.05) (Fig. 5C). Among them, the ratio of gene expression in infected cells within the aviremic section was increased 94- and 98- fold for *SYK* and *CXCL17*, respectively (Fig. 5C). Combining these data, Fig. 5D shows that the gene expression of *CXCL17* and *SYK* was significantly increased (90-100-fold) in infected cells in the aviremic condition as compared with that of the viremic condition. The expression of many other targeted genes, such as *S100A4*, did not change significantly in infected cells upon comparing viremic versus aviremic tissue conditions.

### Measurement of human genes in aviremic donor’s CD4 + T cells

**Fig. 6:**
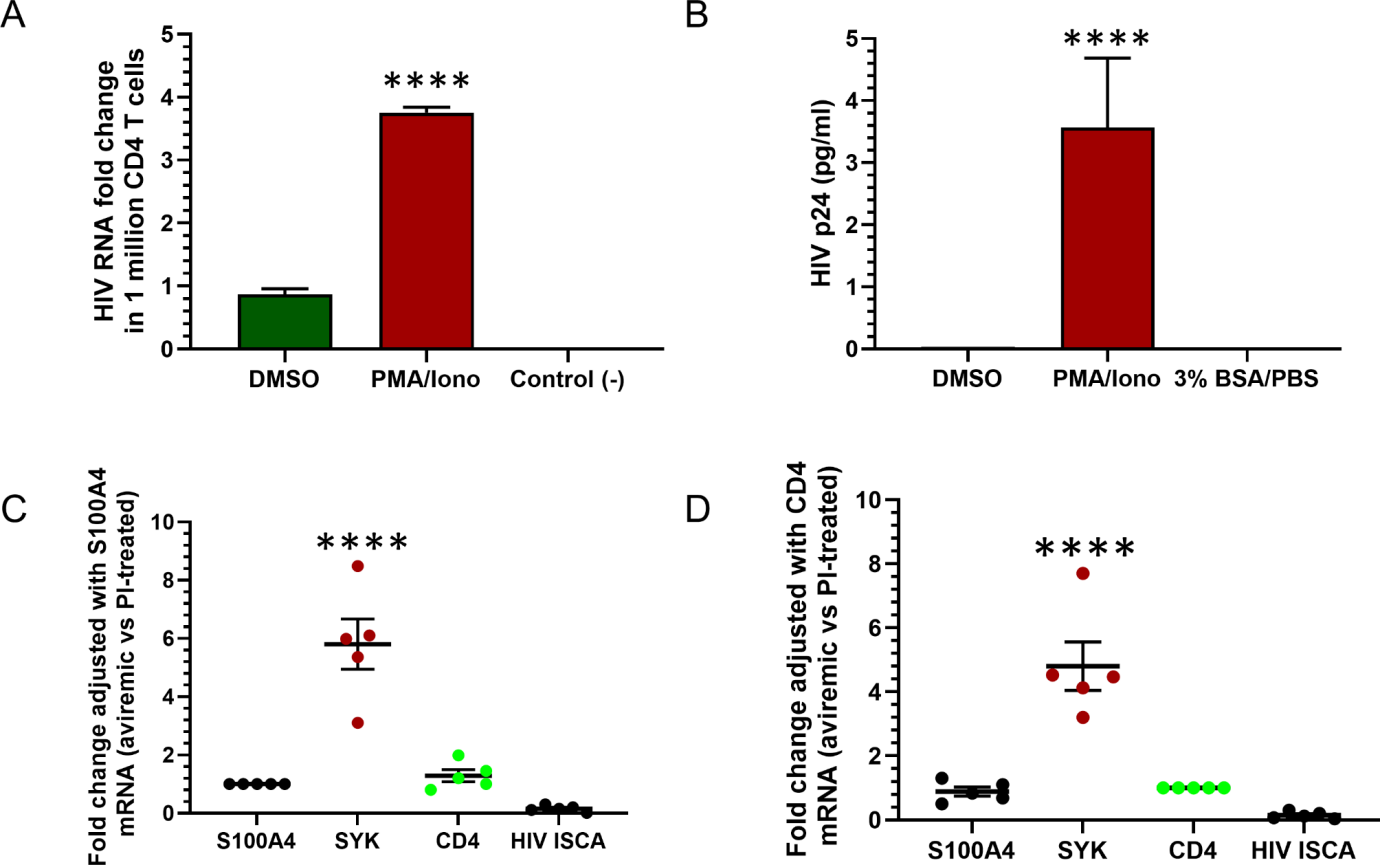
Measurement of SYK gene in CD4 + T cells from aviremic donors: Measured HIV cell-associated RNA **(A)** and HIV p24 protein in culture supernatant **(B)** were significantly increased in PWH donor CD4 + T cells following PMA/Ionomycin stimulation. *SYK* mRNA level was significantly increased in aviremic CD4 + T cells as compared with that of T cells stimulated with PMA/Ionomycin for 48 h after the adjustment of either S100A4 gene **(C)** or *CD4* gene **(D)**

To evaluate whether the changes in gene expression observed in the humanized mouse HIV model were reproducible in people living with HIV (PWH), isolated CD4 + T cells from 5 PWH on ART were used for validation studies using qPCR. First, HIV cell-associated RNA was quantitated from cultured CD4 + T cells with primers/probe targeting the integrase sequence by qPCR and demonstrated a nearly 4- fold increase following PMA/Ionomycin treatment as compared to the DMSO control (Fig. 6A). In addition, HIV p24 protein was also measured in cell culture supernatant following PMA/Ionomycin treatment for 48 h and was found to be significantly higher than that of the DMSO-treated control (Fig. 6B). Both HIV cell-associated viral RNA and culture supernatant p24 data indicated that HIV provirus integrated into CD4 + T cells that were reactivated following PMA/Ionomycin stimulation. Next, *SYK*, *CXCL17*, *S100A4* and *CD4* genes were quantitated in pooled mRNA from aviremic CD4 + T cells with or without PMA/Ionomycin stimulation to simulate a viremic condition in vitro (Fig. 6C&D). The ratio of cellular *SYK* mRNA was 6.5 ± 1.6-fold higher in DMSO treated CD4 + T cells as compared to PMA/Ionomycin treated CD4 + T cells after the adjustment of the S100A4 Ct value (*n* = 5, *p* < 0.0001) (Fig. 6C). Similarly, the ratio of cellular *SYK* mRNA was 4.5 ± 1.1-fold higher in DMSO treated CD4 + T cells compared to PMA/Ionomycin treated CD4 + T cells after adjustment of the *CD4* gene Ct value (*n* = 5, *p* < 0.0001) (Fig. 6D). Neither *S100A4* or *CD4* mRNA changed significantly or demonstrated a significant difference in ratio between DMSO and PMA/Ionomycin treated groups (*n* = 5, *p* > 0.05). The ratio of *CXCL17* mRNA was not changed significantly in isolated CD4 + T cells between DMSO and PMA/Ionomycin treated groups (*p* > 0.05) (data not shown here).

## Discussion

The present study aimed to evaluate the CosMx single-cell molecular imaging (SMI) technology using a humanized HIV mouse model. Mouse spleen FFPE sections from both viremic and aviremic conditions, as determined by the plasma viral load, were evaluated using the CosMx SMI instrument, with staining performed using a 1000-plex panel of human genes along with five custom viral probes. Fit-for-purpose validation revealed a significant difference in both the number of HIV-infected cells in the tissue and the HIV transcripts per infected cells after normalization between viremic and aviremic spleen sections. These qualitative findings indicate that CosMx SMI staining of mouse spleen FFPE sections from the humanized mouse HIV model worked successfully in this study. Additionally, significant changes in the expression of human genes in infected cells were observed when comparing viremic versus aviremic conditions. Notably, the *SYK* gene in HIV-infected CD4 + T cells demonstrated nearly a 100-fold increase for aviremic compared to viremic conditions. This observation was reproduced in isolated CD4 + T cells from five PWH on ART following PMA/ionomycin stimulation in vitro. The mechanism for regulation of SYK during latency is unclear, although several factors such as P‑selectin glycoprotein ligand 1 (PSGL1), toll-like receptors TLR4, TLR7 and mi129-2-3p, have been reported to impact the expression of SYK [29–31]. There is evidence in the literature demonstrating that HIV Nef activates a SYK-dependent cascade that upregulates the PI3K pathway. This cascade results in the downregulation of MHC-I (HLA Class I surface expression), enabling HIV-infected cells to escape immune detection. MHC-I downregulation limits CD8 + T cell-mediated killing and contributes to the pathogenic effects of Nef in infected cells, overall facilitating HIV latency and persistence [32]. Whether this phenomenon exists with other retroviruses during latency is unclear. The elevated expression of *SYK* may be related to the persistence of the HIV reservoir in tissues and could serve as a potential therapeutic target for combating HIV-1. However, further research is needed to explore SYK protein expression in infected host cells and its specific function in this pathway [33]. These findings suggest that CosMx SMI single-cell profiling has the potential to explore novel surrogate biomarkers for host cells in HIV latency.

Despite the effective suppression of HIV replication in patient blood and tissues under ART, residual virus persists in reservoirs within tissues and can reactivate when ART is discontinued [1–3]. These viral reservoirs consist of host cells with integrated HIV provirus DNA in a latent state. The mechanisms underlying their persistence remain unclear and diagnostic biomarkers for this stage of the HIV virus lifecycle are limited. Direct detection of viral RNA and p24 protein from pooled cells or tissue lysate is commonly used to measure the HIV reservoir in blood and tissue [28]. However, this approach is less informative and is hindered by the presence of defective viruses within tissue cells which pose the challenge of distinguishing replication-competent viruses from defective ones. Moreover, information regarding host cell changes following viral infection and during latency is also limited. One potential biomarker of the HIV reservoir is the surface CD32a protein [34], which is predominantly observed in HIV provirus DNA-containing cells and has been proposed as a cell surface marker of the CD4 + T cell HIV reservoir in virally suppressed individuals. However, several studies have failed to reproduce initial findings and suggested that CD32a expression is associated with T-cell activation rather than being a marker of the HIV-1 reservoir [35, 36]. Other potential latency biomarkers include high expression of CD2, CD30 and CD98 on CD4 + T cells [37–39]. While these exploratory biomarkers are exciting, they need further validation for specificity and sensitivity in larger cohort studies. In this preliminary study, our assay strictly relied on NanoString’s validated ∼ 1000 plex probes and did not include either of the forementioned probes except for viral RNA. In this context, exploring novel host cell biomarkers for HIV latency becomes valuable and single-cell molecular profiling of tissue offers novel tools to address this issue.

In recent years, single-cell technology has emerged as a powerful tool for studying disease mechanisms, predicting disease progression, and investigating host-pathogen interactions. Among the various techniques available, 10x Genomics single-cell molecular profiling has been widely utilized for studying isolated single cells across different disease conditions [13, 14]. For single-cell analysis of tissue samples, CosMx SMI (NanoString Technologies) and Xenium (10x Genomics) are two advanced platforms currently available for use. Although their chemistries, panel probe compositions, and the number of probes differ, both platforms offer the capability of single-cell and subcellular mRNA profiling in tissue FFPE sections. Nonetheless, direct comparison of their detection specificity and sensitivity for the same tissue sample remains limited. In this study, a humanized mouse HIV model was generated to evaluate the CosMx SMI technology for proof of concept. The data generated by the CosMx SMI system indicated that it can effectively detect the regulation of multiple genes in tissue sections. The system demonstrated its suitability for 1000-plex human gene expression analysis and an ability to differentiate gene expression following HIV infection and antiviral treatment. These findings highlighted the potential of the CosMx SMI tool for subcellular mRNA profiling of clinical tissue samples.

Despite the quantity and quality of gene expression data collected by the CosMx SMI platform, the current study was limited by the fact that the cells surrounding the individual HIV-infected human cells in this HIV model were murine tissue cells, while the probes used in this study were specific to human host cells. We were therefore unable to utilize an additional advantage of this technology to analyze the spatial profiling changes of surrounding tissue cells [40]. In addition, the study was limited by the use of only one section per spleen which may affect the interpretation of results. Utilizing spatially- separated sections from the same organ could further improve the three- dimensional assessment of gene regulation throughout tissue specimens. We expect to apply spatial profiling using the CosMx SMI technology in future studies involving human tissue sections to explore potential expression patterns or microenvironments that might be present among surrounding HIV-infected cells.

## Conclusions

In conclusion, a humanized mouse HIV model was generated to evaluate the CosMx SMI technology for proof of concept in this study. The data generated by the CosMx SMI system indicated that it can effectively detect the regulation of multiple genes in single cell in tissue sections. The system demonstrated its suitability for multiplex human gene expression analysis and the ability to differentiate gene expression following HIV infection and anti-viral treatment. Our finding that the SYK gene is upregulated in HIV-infected cells in aviremic condition highlights the potential for exploring novel biomarkers in HIV latency. Further study is needed to evaluate spatial transcriptomics in clinical tissue specimens.

## Data Availability

No datasets were generated or analysed during the current study.

## Abbreviations

ART: Antiretroviral Therapy
CXCL17: Chemokine (C-X-C motif) ligand 17
DMSO: Dimethylsulfoxide
FFPE: Formalin-fixed Paraffin-embedded
FOV: Field of View
HIV: Human Immunodeficiency Virus
HNRNPK: Heterogeneous Nuclear Ribonucleoprotein K
IHC: Immunohistochemistry
IL-2: Interleukin-2
ISH: In situ Hybridization
MOI: Multiplicity of infection
PHA: Phytohemagglutinin
PBMC: Peripheral Blood Mononuclear Cells
PFA: Paraformaldehyde
PMA: Phorbol 12-Myristate 13-Acetate
PWH: People living with HIV
qPCR: Quantitative Polymerase Chain Reaction
RAL: Raltegravir
scRNA: Single cell RNA
SCST: Single-cell Spatial Transcriptomics
Simoa: Single Molecule Array
SMI: Spatial Molecular Imager
SYK: Spleen Tyrosine Kinase
UMAP: Uniform Manifold Approximation and Projection
